# Metabolomics Based on ^1^H-NMR Reveal the Regulatory Mechanisms of Dietary Methionine Restriction on Splenic Metabolic Dysfunction in Obese Mice

**DOI:** 10.3390/foods10102439

**Published:** 2021-10-14

**Authors:** Yuhui Yang, Jing Qian, Bowen Li, Manman Lu, Guowei Le, Yanli Xie

**Affiliations:** 1 College of Food Science and Engineering, Henan University of Technology, Zhengzhou 450001, China; yangyuhui1992@126.com (Y.Y.); qianj2021@126.com (J.Q.); lmm199843@163.com (M.L.); 2 State Key Laboratory of Food Science and Technology, School of Food Science and Technology, Jiangnan University, Wuxi 214122, China; lbw1965841356@gmail.com (B.L.); lgw@jiangnan.edu.cn (G.L.)

**Keywords:** methionine restriction, spleen injury, obesity, autophagy, metabolism, metabolomics

## Abstract

Methionine restriction (MR) has been reported to have many beneficial health effects, including stress resistance enhancement and lifespan extension. However, the effects of MR on the splenic metabolic dysfunction induced by obesity in mice remain unknown. This study aimed to investigate the scientific problem and clarify its possible mechanisms. C57BL/6J mice in the control group were fed a control diet (0.86% methionine, 4.2% fat) for 34 weeks, and others were fed a high-fat diet (0.86% methionine, 24% fat) for 10 weeks to establish diet-induced obese (DIO) mouse models. Then, the obtained DIO mice were randomly divided into two groups: the DIO group (DIO diet), the DIO + MR group (0.17% methionine, 24% fat) for 24 weeks. Our results indicated that MR decreased spleen weight, and spleen and plasma lipid profiles, promoted lipid catabolism and fatty acid oxidation, glycolysis and tricarboxylic acid cycle metabolism, and improved mitochondrial function and ATP generation in the spleen. Moreover, MR normalized the splenic redox state and inflammation-related metabolite levels, and increased plasma levels of immunoglobulins. Furthermore, MR increased percent lean mass and splenic crude protein levels, activated the autophagy pathway and elevated nucleotide synthesis to maintain protein synthesis in the spleen. These findings indicate that MR can ameliorate metabolic dysfunction by reducing lipid accumulation, oxidative stress, and inflammation in the spleen, and the mechanism may be the activation of autophagy pathway.

## 1. Introduction

The widespread prevalence of obesity is a worldwide health problem. Increasing evidence suggests that obesity plays a crucial role in the development of metabolic syndrome, type II diabetes, hypertension, fatty liver diseases, cardiovascular diseases, and some cancers [1,2]. Obesity is characterized by chronic low-grade inflammation with permanently increased oxidative stress. Excessive oxidative stress and chronic inflammation induced by fat accumulation damage cellular structures, leading to multiple organ function damage and the emergence of these obesity-related diseases [3]. Past research about obesity has generally concentrated on the liver, heart, kidney, gut, brain, and pancreas; however, the spleen is typically ignored. As the largest lymphoid organ in the human body, the spleen is currently obtaining more attention for the pivotal role played in modulating inflammation, oxidative stress, immune functions, and fat deposition [4,5]. During obesity, increased levels of oxidative stress and inflammation induced by fat deposition lead to apoptosis of splenic cells and splenic damage, ultimately causing immune dysfunction [6]. An increase in oxidative stress and inflammatory response has been demonstrated to be a major mechanism in the pathogenesis and progression of obesity-related spleen diseases [5]. Moreover, previous research has suggested that obesity leads to a decrease in the splenic synthesis of anti-inflammatory factors interleukin-10 (IL-10) by promoting oxidative stress [7]. Interestingly, spleen-derived IL-10 can reduce the inflammation and oxidative stress of multiple organs in the obese state [8,9]. In addition, previous studies on animals and clinical experiences in man have demonstrated that total splenectomy leads to significantly increased plasma total triglyceride (TG), total cholesterol (TC), and low-density lipoprotein cholesterol (LDL-C) levels, significantly decreased plasma high-density lipoprotein cholesterol (HDL-C) levels, and a high rate of atherosclerosis [10,11,12]. Therefore, the findings above imply that alleviation of splenic metabolic dysfunction and function damage may also be a meaningful strategy for preventing obesity and its associated diseases.

Methionine restriction (MR), a dietary regimen that partially deprives the sulfur-containing essential amino acid methionine (Met) from nutrition, extends lifespan in different species (such as yeasts, nematodes, rodents, and human cells) [13,14,15]. In addition, many past studies have consistently indicated that MR also has many beneficial health effects including decline of fat accumulation, improvement of insulin sensitivity, and prevention of obesity, metabolic syndrome, type II diabetes, and some cancers [16,17,18]. MR without calorie restriction can be applied to humans through vegan diets, or/and oral recombinant methioninase (a cloned L-Met depleting enzyme) and intravenous injection of recombinant methioninase [19,20,21]. Recent research has found that MR causes an increase in autophagic capacity by inducing the expression of multiple autophagy-related genes in yeast. Researchers have also further demonstrated that autophagy activation is the basis for the beneficial health effects of MR [22]. Autophagy is a conserved dynamic catabolic process, in which defective protein aggregates and dysfunctional organelles are engulfed, degraded, and recycled, thereby playing an important role in maintaining intracellular homeostasis and metabolic health [23]. Moreover, MR also induces autophagy activation in the liver of mice by reducing the gene expression of the mechanistic target of rapamycin kinase (m-TOR) [24]. Autophagy has been shown to regulate oxidative stress, inflammation, immunity, and lipid metabolism [25,26,27]. Our recent research suggested that MR improves oxidative stress and inflammation in the liver, brain, and gut, and reduces fat accumulation in mice fed a high-fat diet (HFD) [28,29,30]. Previous research has found that MR normalizes a HFD-induced increase in the spleen weight of mice [31]. Furthermore, MR can enhance the tumoricidal capacity of the innate immune system in the tumor microenvironment [32], and can delay immune system aging [33]. However, to date, the effects of MR on splenic metabolite profiles, oxidative stress, inflammation, immunity, and autophagy in obese mice remain unknown.

Based on the above findings, this study aimed to investigate the hypothesis that MR activates the splenic autophagy pathway, which reduces lipid deposition, improves oxidative stress, inflammation, and immunity, and ameliorates splenic function damage in obese mice. To verify this hypothesis, a ^1^H-nuclear magnetic resonance (NMR)-based metabolomics approach combined with traditional biochemical detection and molecular biology methods was employed to explore the effects and mechanisms of MR on obesity-induced spleen metabolic dysfunction in mice.

## 2. Materials and Methods

### 2.1. Animal Experiment

**Figure 1:**
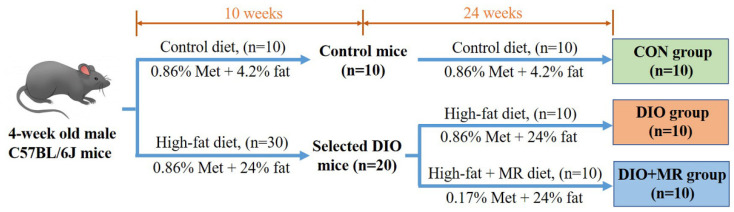
The animal experiment design and schedule of the experiment. Four-week old male C57BL/6J mice (*n* = 40) were first randomly divided into two groups. One group was fed the control diet (*n* = 10, the diet contained 0.86% Met and 4.2% fat) as a control; the other group was fed the high-fat diet (*n* = 30, the diet contained 0.86% Met and 24% fat) to establish diet-induced obese (DIO) mouse models. After 10 weeks of the high-fat diet treatment, given the possibility of the obesity-resistant mice (the lower tertile of body weight, *n* = 10, these mice were excluded from this study and used in an experiment related to obesity resistance) according to our previous study, 20 heavier mice were selected as DIO mice. Then the DIO mice were randomly divided into two groups: (1) the DIO group (*n* = 10), the mice were still fed the same high-fat diet (0.86% Met, 24% fat) for 24 weeks; (2) the DIO + MR group (*n* = 10), the mice were fed a different high-fat diet (0.17% Met, 24% fat) for 24 weeks. The control group (CON group, *n* = 10) mice continued to receive the same control diet (0.86% Met and 4.2% fat) for 24 weeks.

This animal experiment was performed in accordance with the National Guidelines for Experimental Animal Welfare and Experimental Protocol (China, 2006). Forty male C57BL/6J mice (4 weeks old, approximately 20 g) were purchased from the Nanjing Biomedical Research Institute of Nanjing University (Nanjing, China). All the experimental mice were housed under standard laboratory conditions with a temperature of 20–24 °C, relative humidity of 40–60%, 12 h light/12 h dark cycle and ad libitum access to food and water. After acclimatization for 7 days, all mice were first randomly divided into either the control group (*n* = 10, the diet contains 0.86% Met and 4.2% fat) or the HFD group (*n* = 30, the diet contains 0.86% Met and 24% fat) to establish diet-induced obese (DIO) mouse models [34]. Ten weeks later, 20 mice were successfully established into the DIO mouse model. Then the selected DIO mice were randomly divided into two groups: (1) the DIO group (*n* = 10), this group of mice were still fed a same HFD (0.86% Met, 24% fat) for 24 weeks; (2) the DIO + MR group (*n* = 10), this group of mice were fed a different HFD (0.17% Met, 24% fat) for 24 weeks. The control group (CON group, *n* = 10) mice continued receiving the same control diet for 24 weeks. The dosage chosen for MR in this study was based on previous literature [35,36]. The formulas of the three diets are shown in Table S1. The detailed experimental design was shown in Figure 1. The body weight of the mice was recorded weekly. Feed intake at the last week of the study was measured using a monitoring system. Furthermore, body composition (fat mass and lean mass) was detected at the last week using a MesoMR23-060V-I NMR Analyzer (Niumag Co., Ltd., Shanghai, China) with a permanent magnet as previously described [37,38]. NMR Analyzer parameters were as follows: magnetic field strength 0.5 ± 0.08 T, resonance frequency 21.3 MHz, and probe coil diameter 60 mm.

### 2.2. Sample Collection

At the end of this experiment, all mice were fasted overnight, and then sacrificed. Blood samples were collected in Eppendorf tubes containing sodium heparin. After being kept at 4 °C for 30 min, plasma samples were acquired by centrifugation at 3500× *g* and 4 °C for 15 min, and stored at −80 °C for analysis of the levels of plasma lipids, oxidative stress-related indicators, inflammatory factors, and immunoglobulins. Subsequently, spleen tissues were immediately collected, weighed, and then ground using liquid nitrogen and stored at −80 °C for biochemical analysis, nuclear magnetic resonance (NMR) detection, gene expression assays, and Western blot analysis.

### 2.3. Analysis of Blood Glucose, Plasma and Spleen Lipid Parameters, and Spleen Crude Protein

Blood glucose levels were detected using One Touch Sure Step Test Strips (LifeScan, Milpitas, CA, USA). The plasma levels of TG, TC, LDL-C, and HDL-C were tested using analysis kits (Nanjing Jiancheng Bioengineering Institute, Nanjing, China). Spleen lipids were extracted according to a previously reported procedure [39], and the obtained supernatant was used with the aforementioned kits to detect TG and TC levels. The contents of spleen crude protein were analyzed by Kjeldahl determination and calculated using a constant of 6.25.

### 2.4. NMR Spectroscopy and Data Processing

The spleen tissue samples for NMR spectroscopy were prepared according to our previously reported method [28]. All NMR spectra were recorded by Wuhan Zhongke Metaboss Technology Co., Ltd. (Wuhan, China). The detection parameters were programmed according to the published literature [40]. Firstly, the obtained spectra were phase-adjusted and baseline-corrected after referencing to TSP (δ 0.00). Secondly, the signals of spectra were unambiguously assigned to definite metabolites according to the chemical shifts, peaks multiplicities, and relative intensities as in previous literature [41,42,43], and in-house databases. Finally, the spectral regions (δ 0.70–9.00) were subsequently decomposed and integrated into small regions with an equal width of 0.01 ppm. The signals located at δ 4.70–5.20 in the spectra of spleen tissue samples were discarded because of imperfect water suppression. Each bucketed region was then normalized to the tissue wet weight before multivariate statistical analysis.

The data obtained above were imported into the SIMCA-P+ software (version 14.1, Umetrics, Umea, Sweden) for multivariate data analysis [44,45]. Initially, unit variance (UV) scaling was applied for the datasets. Principal component analysis (PCA), an unsupervised pattern recognition method, was employed to identify any trends or outliers in the data. Subsequently, pareto variance (Par) scaling was applied for the data, two supervised pattern recognition methods (partial least-squares discriminant analysis, PLS-DA; orthogonal partial least-squares discriminant analysis, OPLS-DA) were performed to investigate the differentiation of the experimental groups and potential metabolic biomarkers. In the three models, each point on the scores plots represents one sample. Hotelling’s T2 regions presented by ellipses in the score plots of each model define a 95% confidence interval. The calculated parameters of R^2^(cum) and Q^2^(cum) were used to evaluate the quality of PCA, PLS-DA, and OPLS-DA. R^2^(cum) > 0.50 and Q^2^(cum) > 0.50 indicate that the model is robust and has good fitness and prediction [46,47]. The receiver operator characteristic (ROC) curves have been used to evaluate the predictive ability of the PLS-DA and OPLS-DA models. The area under the curve (AUC) of ROC curves was employed to judge the quality of the ROC curve. In addition, cross-validation with *p* values from CV-ANOVA (analysis of variance of the cross-validated residuals) and a permutation test (200 cycles) were performed to evaluate the reliability of the PLS-DA and OPLS-DA models. In the validation plots, the original point values of R^2^ and Q^2^ on the right are higher than all permuted values on the left, and the regression line of Q^2^ values has a trend to intersect the regression line of R^2^ values, indicating that the model is reliable. In order to investigate the metabolites contributing to the separations in different experimental groups, we carried out S-plot under the OPLS-DA model. Meanwhile the variable importance in the projection (VIP) values of all metabolites were obtained to represent their contribution to the discrimination of the two different groups. Finally, VIP values (VIP > 1.00) combined with statistical analysis (SPSS 17.0 software, *p <* 0.05) of metabolites were used to select potential metabolic biomarkers.

### 2.5. Determination of Oxidative Stress Parameters

The spleen and plasma levels of malondialdehyde (MDA), glutathione peroxidase (GSH-Px), reduced glutathione/oxidized glutathione (GSH/GSSG), and total antioxidant capacity (T-AOC) were determined with assay kits (Nanjing Jiancheng Bioengineering Institute, Nanjing, China). The reactive oxygen species (ROS) levels were detected in blood and spleen tissues by a luminol-dependent chemiluminescence assay as described by the previously reported method [48]. The spleen levels of advanced oxidation protein products (AOPPs) were measured using ELISA kit (Xiamen Huijia Bioengineering Institute, Xiamen, China).

### 2.6. Measurement of Inflammatory Cytokines and Immune Parameters

The spleen and plasma levels of interleukin 10 (IL-10), interleukin 6 (IL-6), interleukin 1 beta (IL-1β), tumor necrosis factor alpha (TNF-α), and monocyte chemoattractant protein-1 (MCP-1) were measured by ELISA kits (Xiamen Huijia Bioengineering Co., Ltd., Xiamen, China). The plasma levels of immunoglobulin M (IgM), immunoglobulin G (IgG), and immunoglobulin A (IgA) were tested by ELISA kits (Nanjing Jiancheng Bioengineering Institute, Nanjing, China).

### 2.7. Assays for Spleen Glycogen, ATP/ADP/AMP, Mitochondrial DNA Copy Number, and the Respiratory Chain Enzyme Activities

The spleen levels of glycogen were measured using assay kit (Nanjing Jiancheng Bioengineering Institute, Nanjing, China). The spleen levels of adenosine triphosphate (ATP), adenosine diphosphate (ADP), and adenosine monophosphate (AMP) were measured by high performance liquid chromatography using the commercial kit (Beijing Solarbio Science & Technology Co., Ltd., Beijing, China). Mitochondrial DNA copy number in the spleen was assessed using our previously reported method [17]. The respiratory chain enzyme activities of complex I, complex IV, and complex V in the spleen were analyzed by the commercial kits (Beijing Solarbio Science & Technology Co., Ltd., Beijing, China).

### 2.8. Gene Expression Assays

Total RNA was isolated from spleen tissue samples using a Trizol method. Reverse transcription was carried out using mRNA reverse transcription kits (Vazyme, Suzhou, China). The mRNA expression levels were detected by quantitative real-time polymerase chain reaction (qRT-PCR). The primer sequences of glucose transporter 4 (*GLUT4*), hexokinase2 (*HK2*), phosphate fructose kinase (*PFK*), pyruvate kinase (*PKM*), mitochondrial transcription factor A (*TFAM*), peroxisome proliferator-activated receptor gamma coactivator 1-alpha (*PGC-1α*), mammalian target of rapamycin complex 1 (*mTORC1*), microtubule-associated proteins light chain 3b (*LC3b*), autophagy related genes (*ATG4b*, *ATG5*, *ATG7*, *ATG12*), *Beclin1*, unc-51-like kinase 1 (*ULK1*), lysosomal-associated membrane proteins (*Lamp1*, *Lamp2α*), Gamma-aminobutyric acid receptor-associated protein (*Gabarap*), Gabarap-like 1 (*Gabarapl1*), and β-actin were displayed in Table S2. The mRNA expression levels were expressed as values relative to those of β-actin.

### 2.9. Western Blot Analysis

The procedures of Western blot were operated according to our reported methods [29]. The primary antibodies [Mammalian target of rapamycin (mTOR, #2983, Cell Signaling Technology, Danvers, MA, USA), LC3B (#2775, Cell Signaling Technology), Beclin1 (#3738, Cell Signaling Technology), Sequestosome-1 (p62, #8025, Cell Signaling Technology), and β-actin (A1978, Sigma-Aldrich, Burlington, MA, USA)] and secondary antibody (goat antirabbit IgG, 926-32211, LI-COR) were 1:1000 and 1:10,000 diluted, respectively. The automatic chemiluminescence imaging analysis system was utilized to analyze the bands, and the protein expression levels were shown as values relative to those of β-actin.

### 2.10. Statistical Analysis

All data were statistically analyzed using the SPSS 17.0 software. Differences were compared using one-way ANOVA with a post hoc Tukey’s HSD or Tamhane’s T2. The results were shown as mean ± standard error of the mean (SEM). *p* < 0.05 indicated statistical significance.

## 3. Results

### 3.1. MR Normalized Obesity-Induced Changes of Body Weight and Body Composition

**Figure 2:**
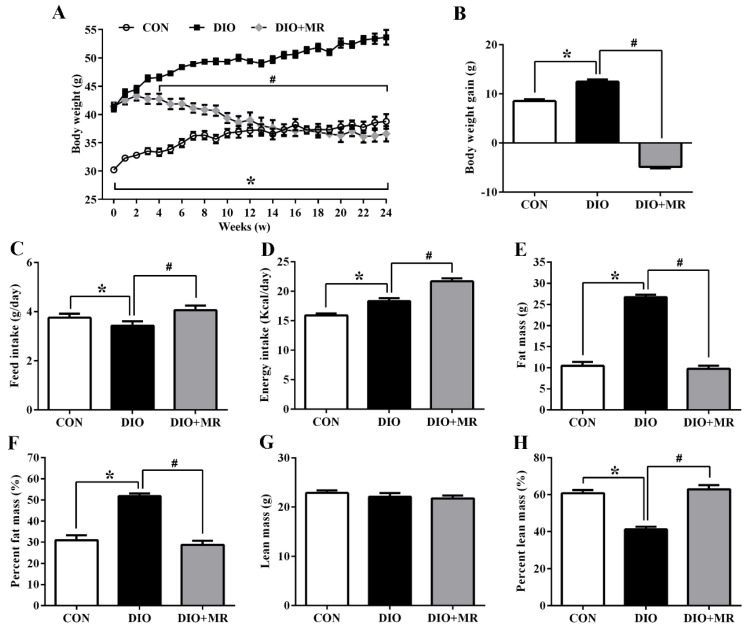
Effects of MR on body weight (**A**), body weight gain (**B**), feed intake (**C**), energy intake (**D**), fat mass (**E**), percent fat mass (**F**), lean mass (**G**), and percent lean mass (**H**) in obese mice. CON, control diet group; DIO, obese + high fat diet group; DIO + MR, obese + high fat with low-methionine diet group. Percent fat mass or percent lean mass = (fat mass/lean mass) ÷ body weight × 100%. All data are shown as mean ± SEM (*n* = 10). * *p* < 0.05 versus CON; ^#^ *p* < 0.05 versus DIO.

As shown in Figure 2A, significant weight differences between the CON mice and the DIO or DIO + MR mice were detected at the beginning of the animal experiment, thereby indicating successful establishment of the DIO mouse models. From the beginning to the end of the experiment, the body weight of the DIO mice significantly increased compared with that of the CON mice, but MR significantly decreased the body weight of mice compared with the DIO mice (*p* < 0.05). In addition, the body weight gain was calculated based on the body weight data at week 24. Compared with the CON mice, the body weight gain was significantly increased in the DIO mice (*p* < 0.05). MR significantly decreased the body weight gain of mice compared with the DIO mice (*p* < 0.05, Figure 2B). In terms of food and energy intake, DIO significantly decreased the feed intake (Figure 2C), and increased the energy intake (Figure 2D) of mice compared with the CON mice (*p* < 0.05). MR significantly increased the feed intake and energy intake of mice compared with the DIO mice (*p* < 0.05). Moreover, the fat mass (Figure 2E) and percent fat mass (Figure 2F) were significantly increased, and the percent lean mass (Figure 2H) was significantly decreased in the DIO mice compared with the CON mice (*p* < 0.05). MR significantly decreased the fat mass and percent fat mass, but significantly increased the percent lean mass of mice compared with the DIO mice (*p* < 0.05). Interestingly, the lean mass had no significant difference in the three experimental groups (*p* > 0.05, Figure 2G).

### 3.2. MR Normalized Obesity-Induced Increases in Spleen Weight, Spleen and Plasma Lipid Levels

**Figure 3:**
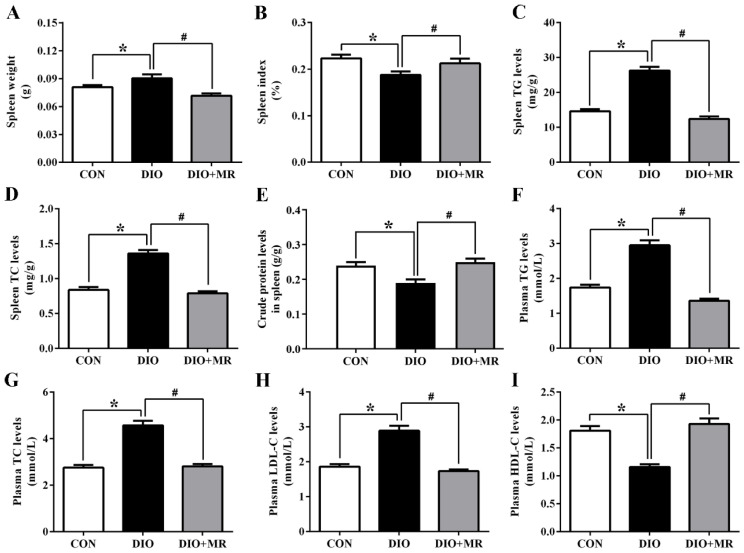
Effects of MR on spleen weight (**A**), spleen index (**B**), spleen TG levels (**C**), spleen TC levels (**D**), splenic crude protein levels (**E**), plasma TG levels (**F**), plasma TC levels (**G**), plasma LDL-C levels (**H**), and plasma HDL-C levels (**I**) in obese mice. TG, triglyceride; TC, total cholesterol; LDL-C, low-density lipoprotein cholesterol; HDL-C, high-density lipoprotein cholesterol. All data are shown as mean ± SEM (*n* = 10). * *p* < 0.05 versus CON; ^#^ *p* < 0.05 versus DIO.

Compared with the CON mice, spleen weight (Figure 3A), spleen TG (Figure 3C) and TC (Figure 3D) levels were significantly increased in the DIO mice (*p* < 0.05). MR significantly decreased the indicators in the DIO + MR mice compared with the DIO mice (*p* < 0.05). Conversely, spleen index (Figure 3B) and spleen crude protein levels (Figure 3E) were significantly decreased in the DIO mice compared with the CON mice, but MR reversed the two changes (*p* < 0.05). Moreover, compared with the CON mice, the DIO mice had significantly increased plasma TG (Figure 3F), TC (Figure 3G), and LDL-C (Figure 3H) levels, and decreased plasma HDL-C (Figure 3I) levels (*p* < 0.05). MR significantly decreased plasma TG, TC, and LDL-C levels, and increased plasma HDL-C levels in the DIO + MR mice compared with the DIO mice (*p* < 0.05).

### 3.3. MR Normalized Obesity-Induced Changes in the Levels of Spleen Metabolites

**Figure 4:**
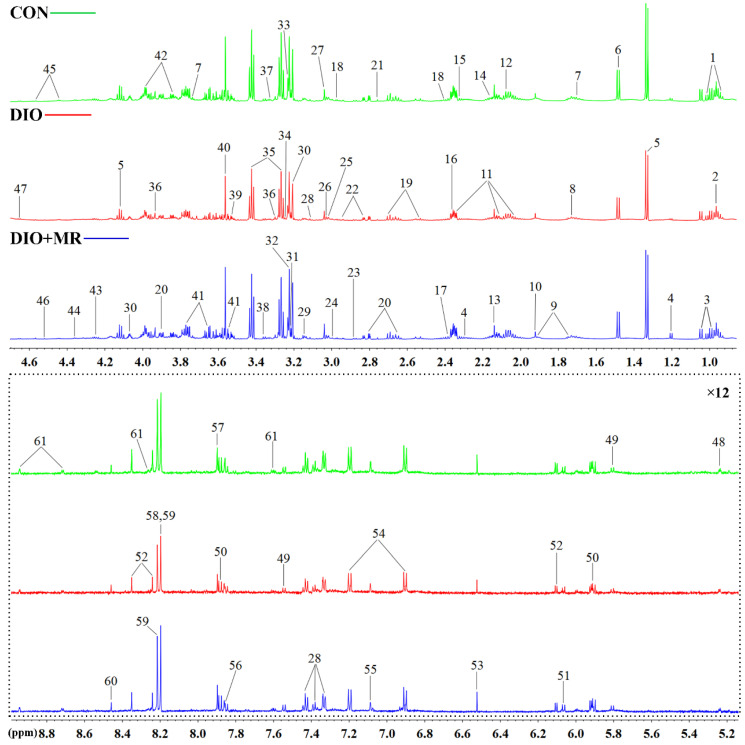
The randomly selected ^1^H-NMR spectra of spleen tissue samples. 1, Isoleucine; 2, 2-Aminobutyrate; 3, Valine; 4, 3-Hydroxybutyrate; 5, Lactate; 6, Alanine; 7, Leucine; 8, Arginine; 9, Lysine; 10, Acetate; 11, Glutamate; 12, Glutamine; 13, Methionine; 14, Glutathione; 15, Acetoacetate; 16, Malate; 17, Pyruvate; 18, 2-Oxoglutarate; 19, Citrate; 20, Aspartate; 21, Sarcosine; 22, Asparagine; 23, Trimethylamine; 24, Histamine; 25, Creatine phosphate; 26, Creatine; 27, Creatinine; 28, Phenylalanine; 29, Ethanolamine; 30, Choline; 31, O-Phosphocholine; 32, O-Phosphoethanolamine; 33, sn-Glycero-3 phosphocholine; 34, Trimethylamine N-oxide; 35, Taurine; 36, Betaine; 37, Tryptophan; 38, Methanol; 39, myo-Inositol; 40, Glycine; 41, Glycerol; 42, Serine; 43, Threonine; 44, ADP; 45, ATP; 46, AMP; 47, β-Glucose; 48, α-Glucose; 49, Uracil; 50, Uridine; 51, Cytidine; 52, Inosine; 53, Fumarate; 54, Tyrosine; 55, Histidine; 56, Benzoate; 57, Xanthine; 58, Oxypurinol; 59, Hypoxanthine; 60, Formate; 61, Niacinamide. The spectra in the dashed box were vertically magnified 12 times (×12). The key information of these metabolites was listed in Table S3.

**Figure 5:**
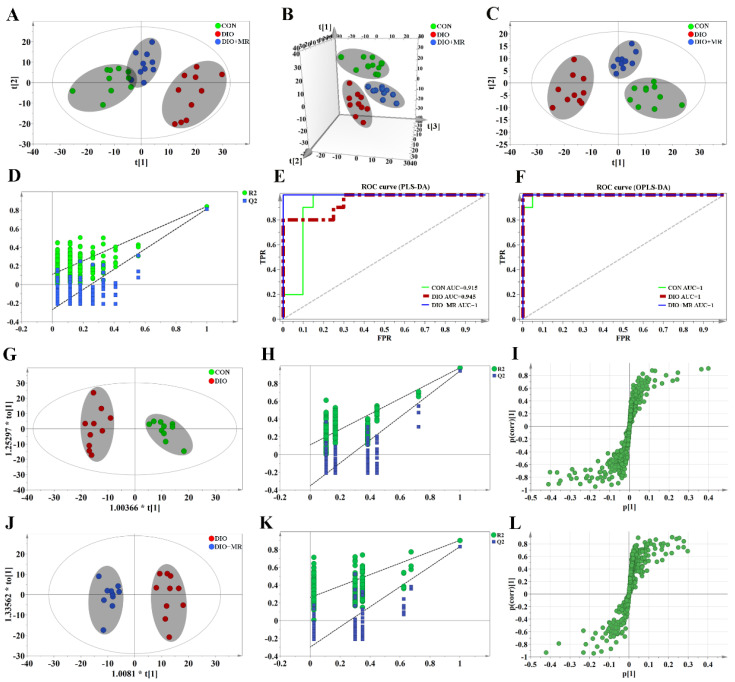
Multivariate statistical analysis based on the ^1^H-NMR spectra of spleen metabolite data acquired from the CON (green circles), DIO (red circles), and DIO + MR (blue circles) mice. (**A**), 2D-PCA score plots (R^2^X = 0.638, Q^2^ = 0.488); (**B**), 3D-PCA score plots (R^2^X = 0.821, Q^2^ = 0.707); (**C**), PLS-DA score plots (R^2^X = 0.570, R^2^Y = 0.758, Q^2^ = 0.548); (**D**), PLS-DA validation plots; (**E**), ROC curve (PLS-DA); (**F**), ROC curve (OPLS-DA); (**G**), OPLS-DA score plots (DIO vs. CON; R^2^X = 0.584, R^2^Y = 0.963, Q^2^ = 0.834); (**H**), OPLS-DA validation plots (DIO vs. CON); (**I**), S-plot (DIO vs. CON); (**J**), OPLS-DA score plots (DIO + MR vs. DIO; R^2^X = 0.608, R^2^Y = 0.934, Q^2^ = 0.812); (**K**), OPLS-DA validation plots (DIO + MR vs. DIO); (**L**), S-plot (DIO + MR vs. DIO).

**Figure 6:**
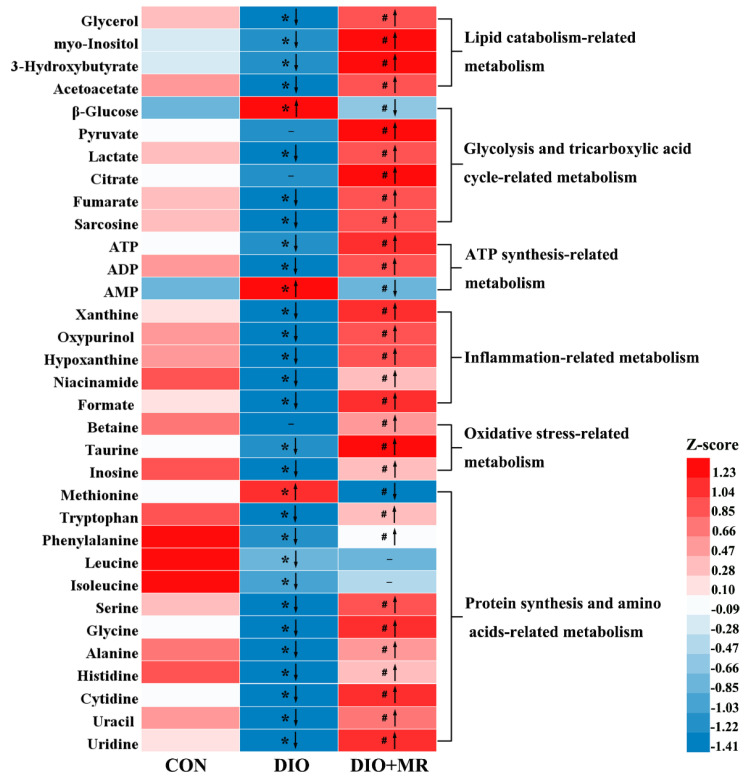
Potential metabolite markers in spleen tissues. The heat map shows normalized relative metabolite levels using the equation Z-score = (value in each sample—average value in all samples)/ (standard deviation of value in all samples). Only when simultaneously meeting *VIP* > 1.00 (variable importance in the projection values) and *p* < 0.05 (one-way ANOVA and Tukey’s test) were the variables ultimately confirmed as potential metabolic biomarkers. “↑/↓” indicate significantly increased/decreased; “-” means insignificant. All data are shown as mean ± SEM (*n* = 10). * *p* < 0.05 versus CON; ^#^ *p* < 0.05 versus DIO.

To explore the effects of MR on the metabolic profiles of spleen tissues, ^1^H-NMR spectroscopy was performed firstly on the spleen tissue samples of mice. Three typical spectra randomly selected from the three experimental groups were shown in Figure 4. Next, 61 metabolites were unambiguously assigned to the signals of the spectra. The detailed information of these identified metabolites was listed in Table S3. The results of multivariate statistical analysis were as follows: a 2D PCA score plot (Figure 5A) showed that the separations from the three experimental groups were no significant difference. However the results of 3D PCA score plot (Figure 5B) indicated that the separations were significant. Furthermore, PLS-DA (Figure 5C) and OPLS-DA (Figure 5G,J) score plots indicated that there were no outliers within the dataset, and separations from the three experimental groups were significant, respectively. All R^2^ and Q^2^ values > 0.50 in the PLS-DA and OPLS-DA models, and all AUC values range from 0.9 to 1 (Figure 5E,F), suggested that the models have good fitness and predictability. Moreover, the validated model of CV-ANOVA (*p* < 0.05) and the permutation tests (Figure 5D,H,K) suggested that the PLS-DA and OPLS-DA models were robust and not overfitting. According to the VIP values (*VIP* > 1) from the S-plot (Figure 5I,L) and *p* values (*p* < 0.05) of assigned metabolites, the potential metabolic biomarkers were displayed in Figure 6.

Compared with the CON mice, the DIO mice had significantly decreased levels of formate, inosine, hypoxanthine, xanthine, oxypurinol, niacinamide, taurine, glycerol, myo-inositol, 3-hydroxybutyrate, acetoacetate, lactate, fumarate, ATP, ADP, tryptophan, phenylalanine, leucine, isoleucine, serine, glycine, alanine, histidine, cytidine, uracil, uridine, and sarcosine, and increased levels of β-glucose, AMP, and Met in spleen tissues. However, the DIO + MR mice had significantly increased levels of formate, inosine, hypoxanthine, xanthine, oxypurinol, niacinamide, betaine, taurine, glycerol, myo-inositol, 3-hydroxybutyrate, acetoacetate, pyruvate, lactate, citrate, fumarate, ATP, ADP, tryptophan, phenylalanine, serine, glycine, alanine, histidine, cytidine, uracil, uridine, and sarcosine, and reduced levels of β-glucose, AMP, and Met in spleen tissues compared with the DIO mice.

### 3.4. MR Counteracted Obesity-Induced Inhibition in Spleen Energy Production

**Figure 7:**
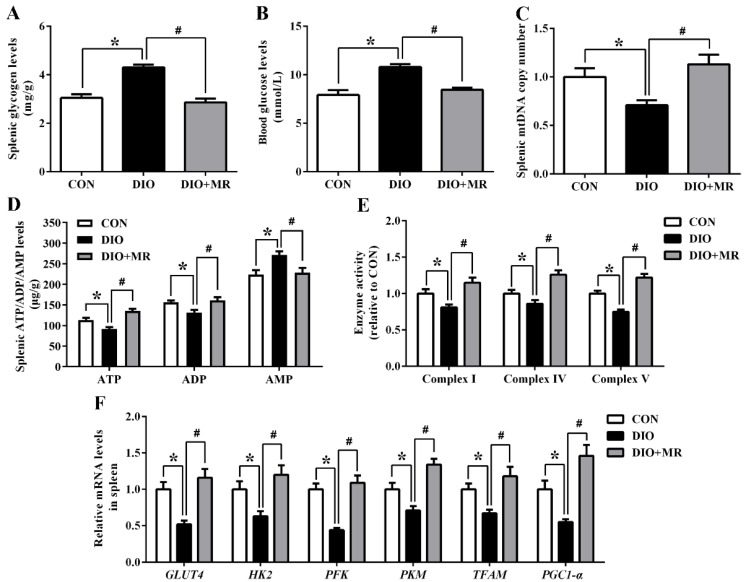
Effects of MR on spleen glycogen levels (**A**), blood glucose levels (**B**), splenic mtDNA copy number (**C**), spleen ATP/ADP/AMP levels (**D**), respiratory chain enzyme activities (**E**), and glucose metabolism-related mRNA expression levels (**F**) in obese mice. ATP, adenosine triphosphate; ADP, adenosine diphosphate; AMP, adenosine monophosphate; *GLUT4*, glucose transporter 4; *HK2*, hexokinase2; *PFK*, phosphate fructose kinase; *PKM*, pyruvate kinase; *TFAM*, mitochondrial transcription factor A; *PGC-1α*, peroxisome proliferator-activated receptor gamma coactivator 1-alpha. All data are shown as mean ± SEM (*n* = 10). * *p* < 0.05 versus CON; ^#^ *p* < 0.05 versus DIO.

Spleen energy production-related parameters were measured to investigate the effects of MR on spleen energy metabolism in obese mice. Spleen glycogen levels (Figure 7A) and blood glucose levels (Figure 7B) were significantly increased in the DIO mice compared with the CON mice, but MR counteracted the two changes in the DIO + MR mice compared with the DIO mice (*p* < 0.05). In addition, compared with the CON mice, the splenic mtDNA copy number (Figure 7C) showed a significant decreasing trend in the DIO mice, but the index was significantly higher in the DIO + MR mice than in the DIO mice (*p* < 0.05). Compared with the CON mice, the DIO mice had significantly reduced spleen ATP and ADP levels, and increased spleen AMP levels (*p* < 0.05, Figure 7D). MR significantly increased spleen ATP and ADP levels, and decreased spleen AMP levels in the DIO + MR mice compared with the DIO mice (*p* < 0.05). Moreover, we found that the activities of respiratory chain enzymes complex I, complex IV, and complex V in spleen were significantly decreased in the DIO mice compared with the CON mice, but the three changes were normalized by MR (*p* < 0.05, Figure 7E). RT-qPCR analysis revealed that *GLUT4*, *HK2*, *PFK*, *PKM*, *TFAM*, and *PGC1-α* mRNA levels were significantly down-regulated in the DIO mice compared with those in the CON mice, but significantly up-regulated in the DIO + MR mice compared with those in the DIO mice (*p* < 0.05, Figure 7F).

### 3.5. MR Reversed Obesity-Induced Enhancement in Spleen Oxidative Stress

Compared with the CON mice, the DIO mice had significantly decreased levels of T-AOC, GSH-Px, and GSH/GSSG, and increased levels of ROS and MDA in spleen tissues and plasma (*p* < 0.05, Table 1). However, the spleen and plasma levels of T-AOC, GSH-Px, and GSH/GSSG were significantly higher, and ROS and MDA levels were significantly lower in the DIO + MR mice than in the DIO mice (*p* < 0.05).

### 3.6. MR Abolished Obesity-Induced Elevation in Spleen Inflammation

The spleen and plasma levels of IL-10 were significantly lower, and IL-6, IL-1β, TNF-α, and MCP-1 levels were significantly higher in the DIO mice than in the CON mice (*p* < 0.05, Table 2). Compared with the DIO mice, the DIO + MR mice had significantly increased levels of IL-10, and decreased levels of IL-6, IL-1β, TNF-α, and MCP-1 in spleen tissues and plasma (*p* < 0.05).

### 3.7. MR Normalized Obesity-Induced Decline in Immune Function

**Figure 8:**
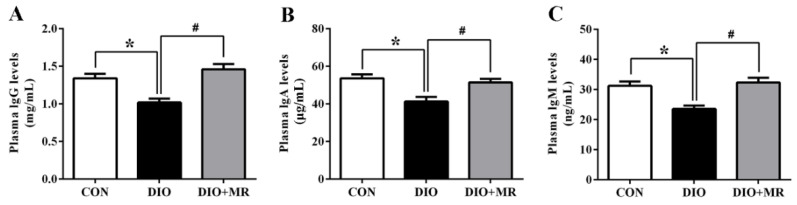
Effects of MR on plasma IgG levels (**A**), plasma IgA levels (**B**), and plasma IgM levels (**C**) in obese mice. IgG, immunoglobulin G; IgA, immunoglobulin A; IgM, immunoglobulin M. All data are shown as mean ± SEM (*n* = 10). * *p* < 0.05 versus CON; ^#^ *p* < 0.05 versus DIO.

To further investigate the effects of MR on immune function, the plasma levels of IgG, IgA, and IgM were measured in mice. As shown in Figure 8, plasma IgG (Figure 8A), IgA (Figure 8B), and IgM (Figure 8C) levels in the DIO mice were significantly decreased compared with those in the CON mice, but plasma IgG, IgA, and IgM levels in the DIO + MR mice were significantly increased compared with those in the DIO mice (*p* < 0.05).

### 3.8. MR Abolished Obesity-Induced Inhibition in Spleen Autophagy Pathway

**Figure 9:**
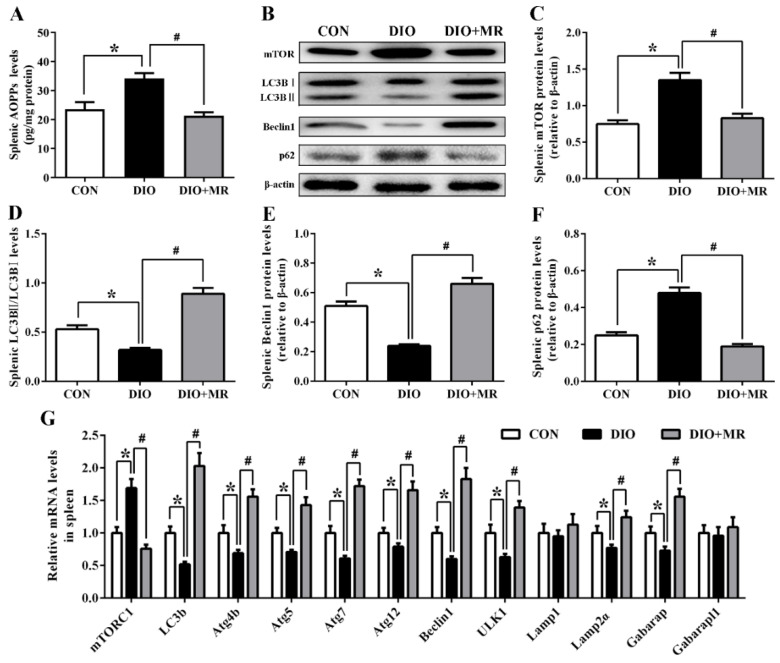
Effects of MR on splenic AOPPs levels (**A**), autophagy-related protein bands (**B**), splenic mTOR protein levels (**C**), splenic LC3BII/LC3BI levels (**D**), splenic Beclin1 protein levels (**E**), splenic p62 protein levels (**F**), and autophagy pathway-related mRNA levels (**G**) in obese mice. AOPPs, advanced oxidation protein products; mTOR, mammalian target of rapamycin; LC3B, Microtubule-associated proteins light chain 3B; p62, sequestosome; mTORC1, mammalian target of rapamycin complex 1; ATG4b/ATG5/ATG7/ATG12, Autophagy related genes; ULK1, unc-51-like kinase 1; Lamp1/Lamp2α, lysosome-associated membrane protein; Gabarap, gamma-aminobutyric acid receptor-associated protein; Gabarapl1, Gabarap-like 1. All data are shown as mean ± SEM (*n* = 10). * *p* < 0.05 versus CON; ^#^ *p* < 0.05 versus DIO.

To investigate potential mechanisms regarding the MR regulation of spleen energy metabolism, oxidative stress, inflammatory response, and immune function, spleen autophagy pathway-related indicators were measured. As shown in Figure 9, compared with the CON mice, obesity induced an increase in spleen AOPPs levels (Figure 9A), elevation in spleen mTOR and p62 protein expression levels (Figure 9A), decline in spleen LC3BII/LC3BI and beclin1 protein expression levels, up-regulation in spleen *mTORC1* gene expression levels (Figure 9B-F), and down-regulation in spleen *LC3B*, *ATG4b*, *ATG5*, *ATG7*, *ATG12*, *beclin1*, *ULK1*, *Lamp2α*, and *Gabarap* gene expression levels in the DIO mice (*p* < 0.05, Figure 9G). However, compared with the DIO mice, MR significantly reduced spleen AOPPs levels, declined spleen mTOR and p62 protein expression levels, elevated spleen LC3BII/LC3BI and beclin1 protein expression levels, down-regulated spleen *mTORC1* gene expression levels, and up-regulated spleen *LC3B*, *ATG4b*, *ATG5*, *ATG7*, *ATG12*, *beclin1*, *ULK1*, *Lamp2α*, and *Gabarap* gene expression levels in the DIO + MR mice (*p* < 0.05).

## 4. Discussion

MR is widely known for its ability to enhance metabolic flexibility, increase stress resistance, and prolong healthy lifespan. In our past research, we found that MR can improve biomarkers of metabolic health in plasma, urine, liver, and gut in mice fed a HFD. In this study, we included body weight, body composition analysis, spleen and plasma lipid levels, splenic systemic metabolite levels, splenic energy production, oxidative stress, inflammation, immune function, and autophagy pathway-related indicators as the analysis variables. Our findings supported the hypothesis that MR can improve splenic oxidative stress and inflammation and ameliorate obesity–splenic metabolic dysfunction in mice, which are likely mediated by activated autophagy.

MR reduced splenic oxidative stress and inflammation and normalized obesity-induced splenic metabolic dysfunction. Previous studies have shown that obesity caused oxidative stress and inflammation, which led to decreased immune function and metabolic dysfunction in spleen [8,49]. In this study, the decreased antioxidant enzyme activities and anti-inflammatory factor levels, increased levels of oxidative damage-related indicators and pro-inflammatory factors, reduced immunoglobulin levels, and disturbed metabolite levels in the spleen of the DIO mice when compared with the CON mice were consistent with this view. Interestingly, we observed that MR significantly increased antioxidant enzyme-related indicators T-AOC, GSH-Px, and GSH/GSSG levels, and reduced oxidative stress damage-related indicators ROS and MDA levels in spleen and plasma, indicating that MR improved splenic oxidative stress in obese mice. The results of splenic metabolome analysis supported this finding. Formate is a precursor of purine synthesis [50]. Previous studies have indicated that circulating formate levels are significantly reduced, and purine synthesis is significantly elevated in highly obese individuals compared with healthy controls [51]. We found that MR significantly increased the levels of formate in the spleen, indicating that MR inhibited splenic purine synthesis. Moreover, purines are broken down in the body to produce uric acid, and xanthine oxidase is a rate-limiting enzyme of uric acid generation from inosine, hypoxanthine, and xanthine in the metabolic process, along with the production of ROS [52,53]. Allopurinol is a standard inhibitor of xanthine oxidase, and has been widely used for treatment of gout caused by high uric acid for decades [54]. In this study, the increased splenic inosine, hypoxanthine, xanthine, and allopurinol levels in the DIO + MR mice compared with the DIO mice implied that MR inhibited the purine metabolism pathway and improved oxidative stress in the spleen. In addition, we observed that MR significantly increased anti-inflammatory factor IL-10 levels, and decreased pro-inflammatory factors IL-6, IL-1β, TNF-α, and MCP-1 levels in spleen and plasma, suggesting that MR reduced splenic inflammation in obese mice. The results of metabolomics analysis also supported this view. Betaine is an important osmoprotectant and has been shown to have anti-inflammatory functions in numerous diseases [55]. Taurine is a free sulfur-containing β-amino acid and plays an important role in the pathogenesis of inflammatory diseases [56]. Niacinamide, the amide form of vitamin B3, has been used as a new alternative treatment for inflammatory diseases [57]. In this study, MR significantly increased the splenic levels of betaine, taurine, and niacinamide, also indicating that MR improved splenic inflammation. The accumulating evidence suggests that improved oxidative stress and inflammation contribute to the repair of splenic function damage [58,59]. Therefore, in the present study, MR ameliorated obesity-induced splenic metabolic dysfunction by improving splenic oxidative stress and inflammation in mice. Supporting this notion, MR abolished obesity-induced increase in spleen weight and decrease in spleen index. This contention is also further supported by the elevated plasma levels of immunoglobulins IgG, IgA, and IgM in the DIO + MR mice.

The finding that MR improved splenic energy metabolism also supports the notion that MR ameliorates obesity-induced splenic metabolic dysfunction. Firstly, MR counteracted obesity-induced inhibition in splenic lipid catabolism. Myo-inositol is a biologically active isomer of inositol and has been found to prevent ectopic fat deposition and decrease free fatty acids in the plasma of mice [60]. Glycerol and free fatty acids are released from the stored TG during the fat mobilization process [61]. Acetoacetate and 3-hydroxybutyrate are the intermediate products of β-oxidation of fatty acids [62]. In this study, MR significantly increased the spleen levels of myo-inositol, glycerol, 3-hydroxybutyrate, and acetoacetate, indicating that MR promoted lipid catabolism in the spleen. This finding is supported by the observed decrease in spleen weight and spleen TG and TC levels, as well as in plasma TG, TC, and LDL-C levels, and the increased plasma HDL-C levels in the DIO + MR mice. Secondly, MR reversed an obesity-induced decline in splenic glycolysis and tricarboxylic acid cycle (TCA) metabolism. This finding is supported by the up-regulated splenic expression of the glycolysis-related genes *GLUT4*, *HK2*, *PFK*, and *PKM* in the DIO + MR mice. Moreover, MR significantly decreased splenic glycogen, β-glucose, and pyruvate, and lactate levels also supported the view. Further supporting this conclusion, MR significantly increased the levels of citrate and fumarate in the spleen, because citrate and fumarate are intermediate metabolites of the TCA metabolism [63]. Thirdly, MR improved splenic mitochondrial function and normalized obesity-induced reduction in splenic energy generation. Mitochondria are the cellular energy-production organelles that convert energy-containing food into ATP using the electron transport chain [64]. In this process, obesity-induced elevation in oxidative stress and inflammation can easily lead to mitochondrial DNA damage and respiratory chain impairment [65]. In this study, we found that MR significantly increased the levels of mtDNA copy number, the activities of respiratory chain enzymes complex I, complex IV, and complex V, and the concentrations of ATP and ADP in the spleen, implying that MR improved mitochondrial function and increased energy generation in the spleen. *TFAM* and *PGC1-α* are important regulators of mtDNA copy number [66]. We observed that MR significantly up-regulated *TFAM* and *PGC1-α* mRNA levels in the spleen, which also supported the notion. Taken together, MR limited ongoing splenic lipid accumulation by increasing splenic energy generation. Subsequently, the generated energy was used to repair and maintain the healthy functions of existing tissues and organs, especially the spleen.

The activation of splenic autophagy pathway is a potential mechanism mediating the aforementioned benefits of MR. Autophagy facilitates the catabolism of nutrients and is essential for regulating nutrients and maintaining cell homeostasis [23]. The most understood pathway of autophagy activation occurs upon inhibition of mTOR by amino acid withdrawal and stress [67]. In our study, dietary MR significantly reduced Met levels and down-regulated protein expression of mTOR and gene expression of mTORC1 in the spleen, implying that MR promoted splenic autophagy. Moreover, our results showed that MR abolished obesity-induced variations in splenic autophagy-related parameters: (1) elevated splenic LC3BII/LC3BI and beclin1 protein expression levels; (2) declined splenic p62 protein expression levels; (3) up-regulated splenic *LC3B*, *ATG4b*, *ATG5*, *ATG7*, *ATG12*, *beclin1*, *ULK1*, *Lamp2α*, and *Gabarap* gene expression levels. AOPPs are a part of protein oxidation markers and have been identified to be negatively related to autophagy [68]. In the present study, MR significantly decreased spleen AOPPs levels, also supporting that MR promoted splenic autophagy. This conclusion is in agreement with that of past studies, suggesting that MR promoted autophagy in yeast and mouse liver [24,69,70]. Interestingly, many studies have shown that autophagy plays a vital role in the process of energy metabolism, oxidative stress, and inflammation [71,72]. Autophagy allows healthy organs to specifically degrade lipid droplets and stimulate β-oxidation, thus decreasing their lipid content [73]. Several previous studies have confirmed that autophagy is markedly impaired under elevated fat deposition [74,75]. HFD-induced obesity leads to increased hepatic fat accumulation, oxidative stress, and inflammation by inhibiting autophagy [72,76]. Autophagy activation caused by dietary intervention reversed this result [77]. Therefore, MR reduced splenic fat accumulation, oxidative stress, and inflammation, likely by promoting splenic autophagy in obese mice.

MR promoted splenic autophagy to maintain protein synthesis, which contributes to the renewal and repair of splenic tissue damage. Autophagy serves as a dynamic recycling system to provide building material and energy for new protein and membrane production to promote survival under conditions of insufficient nutrients [78]. Amino acids are precursors to protein synthesis. Autophagy promotes the degradation of defective intracellular proteins to produce amino acids to maintain protein synthesis [79]. In support of this notion, our study found that MR significantly increased the levels of tryptophan, phenylalanine, serine, glycine, alanine, and histidine in the spleen. This finding is in agreement with that of our past study finding that MR decreased proteolysis-related genes expression, and increased protein synthesis-related genes expression in the gastrocnemius tissues of HFD mice [80]. It is well known that energy is essential for protein synthesis and energy deprivation reduces protein synthesis [81]. Although the amino acids generated by autophagy degradation can also be used to generate ATP for providing energy, the metabolism is relatively weak, and lipids and glycogen are better sources of energy production. Autophagy promotes the degradation of lipids and glycogen to generate free fatty acids and glucose, respectively, which are utilized by mitochondria to generate energy after leaving the lysosomal cavity [82]. In our study, MR significantly increased feed intake and energy intake, decreased body weight and body weight gain, fat mass and percent fat mass, splenic lipid and glycogen levels, and promoted splenic lipid catabolism and glycolysis, increasing ATP generation. Moreover, MR also improved splenic intracellular homeostasis by reducing oxidative stress and inflammation, which provided basic conditions for protein synthesis. Furthermore, we found that MR significantly increased the levels of sarcosine, cytidine, uridine, and uracil in the spleen. Sarcosine, an endogenous amino acid, participates in one-carbon metabolism to activate single carbons for nucleotide synthesis and protein synthesis [83]. Cytidine, uridine, and uracil are the precursors of nucleotide synthesis, which can enhance protein synthesis and cell proliferation [84]. These findings all supported that MR promoted autophagy to maintain protein synthesis and ameliorate metabolic dysfunction in the spleen of obese mice. The conclusions are also indirectly supported by the fact that MR did not significantly reduce lean mass. Even more interesting is that instead, MR significantly increased the lean mass percentage and splenic crude protein levels in our current study.

## 5. Conclusions

In summary, our results first suggested that MR improved splenic oxidative stress and inflammation, and increased immunoglobulin production in obese mice. Moreover, MR reduced splenic lipid accumulation by promoting lipid catabolism, glycolysis and TCA metabolism, and improving mitochondrial function. Furthermore, MR activated the splenic autophagy pathway, which may contribute to the beneficial effects. Therefore, MR ameliorated obesity-induced splenic metabolic dysfunction, likely by promoting autophagy in mice. Our study developed the new idea that dietary MR intervention may be an effective strategy for preventing or treating obesity-related splenic metabolic diseases.

## Figures and Tables

**Table 1:** Effects of MR on the levels of oxidative stress-related parameters in spleen and plasma in obese mice.

Indicators	CON	DIO	DIO + MR
Spleen			
T-AOC (U/mg protein)	0.86 ± 0.05	0.49 ± 0.03 *	0.83 ± 0.06 ^#^
GSH-Px (U/mg protein)	70.13 ± 2.96	51.64 ± 2.33 *	73.20 ± 3.44 ^#^
GSH/GSSG	1.25 ± 0.06	0.89 ± 0.02 *	1.30 ± 0.05 ^#^
ROS (10^3^ cd/mg)	35.04 ± 1.67	57.32 ± 2.45 ^#^	38.36 ± 2.02 *
MDA (nmol/mg protein)	2.24 ± 0.12	3.16 ± 0.10 *	2.18 ± 0.11 ^#^
Plasma			
T-AOC (U/mL)	3.76 ± 0.17	2.57 ± 0.11 *	3.82 ± 0.20 ^#^
GSH-Px (U/mL)	73.51 ± 3.49	51.73 ± 2.85 *	75.69 ± 4.02 ^#^
GSH/GSSG	0.32 ± 0.02	0.25 ± 0.01 *	0.34 ± 0.02 ^#^
ROS (10^3^ cd/μL)	31.26 ± 2.03	46.89 ± 2.69 *	29.77 ± 2.30 ^#^
MDA (nmol/mL)	13.06 ± 0.81	19.22 ± 0.73 *	14.11 ± 0.71 ^#^

T-AOC, total antioxidant capacity; GSH-Px, glutathione peroxidase; GSH/GSSG, reduced glutathione/oxidized glutathione; ROS, reactive oxygen species; MDA, malondialdehyde. All data are shown as mean ± SEM (*n* = 10). * *p* < 0.05 versus CON; ^#^ *p* < 0.05 versus DIO.

**Table 2:** Effects of MR on the levels of inflammatory cytokines in spleen and plasma in obese mice.

Indicators	CON	DIO	DIO + MR
Spleen			
IL-10 (pg/mg protein)	52.03 ± 2.11	34.04 ± 1.55 *	54.73 ± 2.68 ^#^
IL-6 (pg/mg protein)	32.25 ± 1.30	39.12 ± 1.46 *	33.45 ± 1.19 ^#^
IL-1β (pg/mg protein)	46.81 ± 2.12	57.69 ± 1.89 *	48.33 ± 1.75 ^#^
TNF-α (pg/mg protein)	63.12 ± 2.70	84.56 ± 3.23 *	66.72 ± 2.16 ^#^
MCP-1 (pg/mg protein)	24.66 ± 1.08	32.98 ± 1.24 *	22.21 ± 1.00 ^#^
Plasma			
IL-10 (pg/mL)	145.60 ± 6.52	117.14 ± 5.07 *	138.32 ± 5.86 ^#^
IL-6 (pg/mL)	58.62 ± 1.83	73.09 ± 2.35 *	53.71 ± 1.77 ^#^
IL-1β (pg/mL)	67.53 ± 2.16	91.80 ± 2.54 *	69.64 ± 2.09 ^#^
TNF-α (pg/mL)	116.24 ± 4.05	150.67 ± 5.46 *	121.31 ± 3.13 ^#^
MCP-1 (pg/mL)	36.44 ± 1.20	49.79 ± 1.94 *	37.08 ± 1.69 ^#^

IL-10, interleukin 10; IL-6, interleukin 6; IL-1β, interleukin 1 beta; TNF-α, tumor necrosis factor alpha; monocyte chemoattractant protein-1 (MCP-1). All data are shown as mean ± SEM (*n* = 10). * *p* < 0.05 versus CON; ^#^ *p* < 0.05 versus DIO.

## Data Availability

All data are contained within the article and Supplementary File.

## Supplementary Materials

The following are available online at https://www.mdpi.com/article/10.3390/foods10102439/s1, Table S1: The ingredients of the experimental diets (g/100 g of diet); Table S2: Sequences of primers used in quantitative real-time reverse transcription PCR; Table S3: ^1^H chemical shift assignment of the metabolites in the spleen of mice.
