# Supplementary material for: Metabolomics Based on 1H-NMR Reveal the Regulatory Mechanisms of Dietary Methionine Restriction on Splenic Metabolic Dysfunction in Obese Mice
Source: Foods. 2021 Oct 14;10(10):2439. doi: 10.3390/foods10102439 (PMC8535630; doi:10.3390/foods10102439)
Supplement: Supplementary file 1 [file foods-10-02439-s001.zip › foods-1397297-supplementary.pdf]

Supporting information

Table S1. The ingredients of the experimental diets (g/100 g of diet)

Ingredient	CON	DIO	DIO+MR
Soy protein [1]	7.59	7.59	7.59
L-Arginine	0.66	0.66	0.66
L-Histidine	0.18	0.18	0.18
L-Isoleucine	0.51	0.51	0.51
L-Leucine	0.64	0.64	0.64
L-Lysine	0.11	0.11	0.11
L-Methionine [2]	0.69	0.69	0.00
L-Phenylalanine	0.85	0.85	0.85
L-Threonine	0.65	0.65	0.65
L-Tryptophan	0.10	0.10	0.10
L-Valine	0.57	0.57	0.57
L-Glutamic acid [2]	1.37	1.37	2.06
L-Glycine	2.08	2.08	2.08
Corn starch	64.09	44.29	44.29
maltodextrin	5.00	5.00	5.00
Sucrose	0.10	0.10	0.10
Soybean oil	2.00	2.00	2.00
Pork Lard	2.20	22.00	22.00
Cellulose	5.00	5.00	5.00
Mineral mixture-AIN-76A	3.50	3.50	3.50
Mineral vitamin-AIN-76A	1.00	1.00	1.00
Choline chloride	0.11	0.11	0.11
CMC	1.00	1.00	1.00
Total	100.00	100.00	100.00

CON, control diet group; DIO, obese + high fat diet group; DIO + MR, obese + high fat with low-methionine diet group. [1] Amino acid composition of soy protein were as follows: 6.19% leucine, 4.11% isoleucine, 5.49% valine, 1.18% methionine, 1.66% cysteine, 4.09% phenylalanine, 2.57% tyrosine, 4.83% lysine, 2.21% threonine, 1.07% tryptophan, 1.99% histidine, 6.11% arginine, 3.30% serine, 3.25% alanine, 5.56% proline, 3.27% glycine, 17.49% glutamic acid 9.44% aspartic acid. 1 g cysteine is equal to 0.64 g methionine. [2] When the methionine content in the diet was decreased, the glutamic acid was increased to compensate for the reduced methionine content and to create equal amounts of total amino acids.

Table S2. Sequences of primers used in quantitative real-time reverse transcription PCR

Gene name	Forward primer (5′–3′)	Reverse primer (5′–3′)
GLUT4	CCTTTGCACACGGCTTCCGA	TGTTCAATCACCTTCTGTGGGGCA
HK2	TGCTGCCGACCTTTGTGA	AAGGTCCAGAGCCAGGAACTC
PFK	TGTGGTCCGAGTTGGTATCTT	GCACTTCCAATCACTGTGCC
PKM	AGGGGCACCCAAGTACATC	TGCCGGAGGAAAGTGAATGAC
TFAM	ATCCCCTCGTCTATCAGTCTTGTCT	TTCTGCTTCTGGTAGCTCCCTC
PGC-1α	CAAGCCAAACCAACAACTTTATCTC	AAGCCTTGAAAGGGTTATCTTGGT
mTORC1	AGGAACTAGAGGTAGCTGCGATTAA	GAGTGGTGAGGCAGGATGTGAA
LC3b	CCCACCAAGATCCCAGTGAT	CCAGGAACTTGGTCTTGTCCA
ATG4b	CATCCATCAGATAGCGCAAA	TGATTTCCTCCATCACCACA
ATG5	GACAGATTTGACCAGTTTTGGGC	GGGTTTCCAGCATTGGCTCTATC
ATG7	TGCCTATGATGATCTGTGTC	CACCAACTGTTATCTTTGTCC
ATG12	GGCCTCGGAACAGTTGTTTA	CAGCACCGAAATGTCTCTGA
Beclin1	GGAAAAGAACCGCAAGGTGGTG	AAACTGTCCGCTGTGCCAGATG
ULK1	GCTCCGGTGACTTACAAAGCTG	GCTGACTCCAAGCCAAAGCA
Lamp1	CTCTGCCTCCTTTCTGACCA	GCAGGGAAATGTTCACGAT
Lamp2α	GATGTGCCTCTCTCCGGTTA	ATTGGACTGAACGGCTCCTA
Gabarap	AAGAGGAGCATCCGTTCGAGA	GCTTTGGGGGCTTTTTCCAC
Gabarapl1	GGACCACCCCTTCGAGTATC	CCTCTTATCCAGATCAGGGACC
β-actin	GGGTCAGAAGGACTCCTATG	GTAACAATGCCATGTTCAAT

GLUT4, glucose transporter 4; HK2, hexokinase2; PFK, phosphate fructose kinase; PKM, pyruvate kinase; TFAM, mitochondrial transcription factor A; PGC-1α, peroxisome proliferator-activated receptor gamma coactivator 1-alpha; mTORC1, mammalian target of rapamycin complex 1; LC3b, microtubule-associated proteins light chain 3b; ATG4b, ATG5, ATG7, ATG12, autophagy related genes; ULK1, unc-51-like kinase 1; Lamp1, Lamp2α, lysosome assoc membrane proteins; Gabarap, gamma-aminobutyric acid receptor-associated protein; Gabarapl1, Gabarap-like 1.

Table S3. ^{1}H chemical shift assignment of the metabolites in the spleen of mice

Keys	Metabolites	Moieties	δ ^{1}H (ppm) and multiplicity
1	Isoleucine	αCH, βCH, $βCH_3$, $γCH_2$, $δCH_3$	3.68(d), 1.99(m), 1.01(d), 1.26(m), 1.47(m), 0.94(t)
2	2-Aminobutyrate	$βCH_3$, $αCH_2$, O-CH_3	0.96(t), 1.91(m), 3.68(t)
3	Valine	$αCH_3$, βCH, $γCH_3$	3.62(d), 2.28(m), 0.99(d), 1.04(d)
4	3-Hydroxybutyrate	$γCH_3$, $αCH_2$, βCH	1.20(d), 2.28(dd), 2.42(dd), 4.16(m)
5	Lactate	$βCH_3$, αCH	1.33(d), 4.12(q)
6	Alanine	$βCH_3$, αCH	1.48(d), 3.77(q)
7	Leucine	αCH, $βCH_2$, γCH, $δCH_3$	3.73(t), 1.72(m), 0.96(d), 0.91(d)
8	Arginine	$γCH_2$, $βCH_2$, $δCH_2$, αCH	1.73(m), 1.93(m), 3.23(t), 3.75(t)
9	Lysine	αCH, $βCH_2$, $γCH_2$, $δCH_2$	3.77(t), 1.89(m), 1.74(m)
10	Acetate	CH_3	1.92(s)
11	Glutamate	αCH, $βCH_2$, $γCH_2$	2.05(m), 2.12(m), 2.35(m), 3.75(m)
12	Glutamine	αCH, $βCH_2$, $γCH_2$	3.68(t), 2.10(m), 2.15(m), 2.45(m)
13	Methionine	αCH, $βCH_2$, $γCH_2$, S-CH_3	3.87(t), 2.11(m), 2.65(t), 2.14(s)
14	Glutathione	αCH, $αCH_2$, $βCH_2$, $γCH_2$	2.16(m), 2.57(m), 2.95(dd), 4.58(m)
15	Acetoacetate	CH_3	2.32(s)
16	Malate	$βCH_2$, $β'CH_2$, αCH	2.36(dd), 2.67(dd), 4.31(m)
17	Pyruvate	CH_3	2.38(s)
18	2-Oxoglutarate	$αCH_2$, $βCH_2$	2.41(t), 2.98(t)
19	Citrate	CH_2	2.55(d), 2.68(d)
20	Aspartate	CH_2, -CH-NH_2	2.67(dd), 2.81(dd), 3.9(dd)
21	Sarcosine	CH_3, CH_2	2.76(s), 3.65(s)
22	Asparagine	CH_2	2.83(dd), 2.95(dd)
23	Trimethylamine	CH_3	2.88(s)
24	Histamine	CH_2, CH, N-CH=N	3.00(t), 3.29(t), 6.8(s), 7.97(s)
25	Creatine phosphate	N-CH_3, CH_2	3.01(s), 3.94(s)
26	Creatine	CH_3, CH_2	3.03(s), 3.93(s)
27	Creatinine	CH_3, CH_2	3.04(s), 4.05(s)
28	Phenylalanine	2,6-CH, 3,5-CH, 4-CH	3.11(d), 7.32 (m), 7.42 (m), 7.37 (m)
29	Ethanolamine	CH_2-NH_2, CH_2-OH	3.13(t), 3.83(t)
30	Choline	O-CH_2, N-CH_2, N-$(CH_3)_3$	4.07(t), 3.53(t), 3.20(s)
31	O-Phosphocholine	O-CH_2, N-CH_2, N-$(CH_3)_3$	3.21(s), 3.57(m), 4.17(m)
32	O-Phosphoethanolamine	O-CH_2, CH_2-NH_2	3.22(m), 3.98(m)
33	sn-Glycero-3 phosphocholine	CH, CH_2, O-CH_2, N-CH_2, N-$(CH_3)_3$	3.23(s), 3.6(dd), 3.67(m), 3.68(dd), 3.86(m), 3.92(m), 3.95(m), 4.32(m)
34	Trimethylamine N-oxide	CH_3	3.24(s)
35	Taurine	-CH_2-S, -CH_2-NH_2	3.27(t), 3.43(t)
36	Betaine	CH_3, CH_2	3.30(s), 3.94(s)
37	Tryptophan	$βCH_2$, $β'CH_2$, αCH, 5CH, 6CH, 2CH, 7CH, 4CH	3.31(dd), 3.49(dd), 4.06(dd), 7.2 (t), 7.27(t), 7.30(s), 7.55(d), 7.73(d)
38	Methanol	CH_3	3.36(s)
39	myo-Inositol	5-CH, 4,6-CH, 2-CH	3.28(t), 3.53(dd), 3.62(t), 4.06(m)
40	Glycine	CH_2	3.56(s)
41	Glycerol	CH, CH_2	3.56(dd), 3.65(dd), 3.78(m)
42	Serine	-CH-NH_2, CH_2	3.84(dd), 3.95(dd), 3.99(dd)
43	Threonine	αCH, βCH, $γCH_3$	1.32(d), 4.25(m), 3.58(d)

44	ADP	N-CH-N, N-CH=N, C-NH_2, N-CH, CH-OH, CH, CH_2	4.2(m), 4.37(m), 4.57(dd), 4.74(dd), 6.13(dd), 8.27(s), 8.58(s)
45	ATP	N-CH-N, N-CH=N, C-NH_2, N-CH, CH-OH, CH, CH_2	4.2(m), 4.44(m), 4.57(dd), 4.74(dd), 6.15(d), 8.23(s), 8.38(s)
46	AMP	N-CH-N, N-CH=N, N-CH, CH-OH, CH, CH_2	4.01(m), 4.36(m), 4.49(m), 4.78(dd), 6.11(d), 8.24(s), 8.62(s)
47	β-Glucose	1-CH, 2-CH, 3-CH, 4-CH, 5-CH, 6-CH	4.65(d), 3.25(dd), 3.49(t), 3.41(dd), 3.46(m), 3.73(dd), 3.90(dd)
48	α-Glucose	1-CH, 2-CH, 3-CH, 4-CH, 5-CH, 6-CH	5.24(d), 3.54(dd), 3.71(dd), 3.42(dd), 3.84(m), 3.78(m)
49	Uracil	5-CH, 6-CH	5.8(d), 7.53(d)
50	Uridine	2-CH, 5-CH, 1-CH, 6-CH	4.36(t), 5.9(d), 5.92(d), 7.88(d)
51	Cytidine	1-CH, 5-CH, 6-CH	5.92(d), 6.06(d), 7.85(d)
52	Inosine	3-CH, 1-CH, 8-CH, 2-CH	4.44(dd), 6.11(d), 8.24(s), 8.35(s)
53	Fumarate	CH, CH_3	6.52(s)
54	Tyrosine	2,6-CH, 3,5-CH	7.19(dd), 6.90(d)
55	Histidine	αCH, βCH_2	7.88(s), 7.09(s)
56	Benzoate	3,4,5-CH, 2,6-CH	7.48(m), 7.60(m), 7.86(m)
57	Xanthine	CH	7.90(s)
58	Oxypurinol	N-CH	8.19(s)
59	Hypoxanthine	N-CH, CH	8.19(s), 8.21(s)
60	Formate	CH	8.46(s)
61	Niacinamide	O=C-NH_2, 5-CH, 4-CH, NH-CH	7.6(dd), 8.26(m), 8.72(m), 8.94(d)

s, singlet; d, doublet; t, triplet; q, quartet; dd, doublet of doublets; m, multiplet; ATP, adenosine triphosphate; ADP, adenosine diphosphate; AMP, adenosine monophosphate.
